# Study of the effect of pain on postoperative rehabilitation of patients with uterine malignant tumor

**DOI:** 10.3389/fsurg.2022.1052800

**Published:** 2023-01-04

**Authors:** Xiaohong Lv, Chunlai Li, Min Tang, Dan Yuan, Yu Zhong, Yubo Xie

**Affiliations:** ^1^ Department of Anesthesiology, The First Affiliated Hospital of Guangxi Medical University, Guangxi, China; ^2^ Guangxi key Laboratory of Enhanced Recovery after Surgery for Gastrointestinal Cancer, The First Affiliated Hospital of Guangxi Medical University, Guangxi, China; ^3^ Department of Anesthesiology, The Second Affiliated Hospital of Guilin Medical College, Guangxi, China

**Keywords:** patients with uterine malignant tumor, surgery, acute postoperative pain, health-related quality of life, postoperative rehabilitation

## Abstract

**Objective:**

The relationship between acute postoperative pain (APSP) and health-related quality of life (HRQoL) in patients with uterine malignant tumor after operation was evaluated with self-rating scales, and the influencing factors of postoperative rehabilitation were screened.

**Methods:**

A total of 102 patients undergoing elective surgery for Gynecology in the First Affiliated Hospital of Guangxi Medical University were included in this study. PCS, SAS, NRS and EQ-5D scales were evaluated 1 day before surgery, and NRS and EQ-5D scales were evaluated 1,3,7,14, and 30 days after surgery. In addition, the general and perioperative information of patients was collected from the medical record system of the hospital.

**Results:**

From the 1st to the 30th day after operation, the NRS and EQ-5D-5L scores of patients decreased gradually, and EQ-VAS scores increased gradually. NRS score was correlated with EQ-5D score (*P* < 0.01). Postoperative hospital stay, Education level, PCS score and NRS score (Overall state and Active state) were the principal influencing factors of EQ-5D score (*P* < 0.05). Patients in the pain group had a later time to get out of bed and eat, a higher incidence of postoperative complications, and a longer postoperative hospital stay (*P* < 0.05). Endoscopic surgery can reduce postoperative pain and promote postoperative rehabilitation (*χ*^2 ^= 37.631, *P* < 0.001).

**Conclusions:**

The postoperative rehabilitation of patients in the pain group was poor. Minimally invasive surgery can reduce postoperative pain and promote postoperative rehabilitation. EQ-5D score can be used as a subjective index to evaluate postoperative rehabilitation.

**Trial Registration:**

Chinese Clinical Trial Registry (identifier: ChiCTR2000032759).

## Introduction

With the development of society, people's living standards are increasing, and medical technology is also improving. In addition to the treatment of diseases, people have put forward higher requirements for the medical system, comfortable medical treatment is imminent. In the past clinical work, we always evaluated the patients' postoperative rehabilitation by objective indexes, such as postoperative hospital stay, and ignored the patients' subjective feelings (1, 2). Therefore, we should integrate the objective and subjective indicators of patients to evaluate their postoperative rehabilitation, and constantly improve their diagnosis and treatment plans to promote their recovery.

With the improvement of medical technology, many diseases, especially tumors, can be removed surgically. This not only prolongs the patient's life, but also increases the incidence of postoperative pain (3). Studies have shown that the incidence of acute postoperative pain (APSP) can be as high as 80% (4, 5). Pain is an unpleasant feeling, usually accompanied by painful psychological and emotional feelings, caused by stimuli that cause or may cause tissue damage. Pain also transmits harmful stimuli to the central nervous system, causing neuroendocrine stress reactions. It not only stimulates the sympathetic nervous system, but also affects the blood coagulation system, cardiovascular system, respiratory system, immune system, digestive system, etc. It can cause blood hypercoagulability, myocardial ischemia, pulmonary complications, infection, nausea and vomiting and other adverse reactions (6). If not well controlled, APSP can seriously affect the physical and mental health of patients, delay recovery, increase hospital stay and medical costs, and turn into chronic pain, causing long-term pain to patients (7–10).

To investigate the subjective and objective effects of pain on postoperative recovery, we established a double-standard evaluation system by taking advantage of two well established evaluation protocols, Number Rating Scale (NRS) and EuroQol-5 dimension (EQ-5D), as the standard to evaluate the APSP and health-related quality of life (HRQoL) at day 1 before and day 1, 3, 7, 14, and 30 after surgery, respectively. The double-standard evaluation system allowed us to test the applicability of the two evaluation protocols not only by the law of natural postoperative rehabilitation along with the time, but also by a mutually confirm manner, for example the low level of APSP always come with high level of HRQoL.

NRS is a widely used evaluation protocol in the clinical evaluation of APSP in China. This scale is composed of 11 points from 0 to 10, the number from low to high indicates from no pain to the most pain, 0 points means no pain, 10 points means severe pain, and patients choose different points to quantify the pain (11, 12). The EQ-5D scale is mainly composed of a health status description system (EQ-5D-5L) and a health-related overall quality of life indicator (EQ-VAS) (13–16). In this study, EQ-5D-5L was selected as the health status description system. It has been shown that EQ-5D-5L can completely reflect the slight differences between patients with different health conditions, and there is no severe ceiling effect. EQ-5D-5L has a total of five dimensions, namely mobility, self-care, daily activities, pain or discomfort, and anxiety or depression. Each dimension is rated as not difficult, somewhat difficult, moderately difficult, severely difficult, and very difficult. Each of the five-dimension levels corresponds to a score of 1 to 5. The lower the score, the better the health ability assessment. EQ-VAS scores range from 0 to 100, with 100 representing the best health and 0 representing the worst. Both protocols are simple, easy to understand, and sensitive.

The existing detailed medical record system has documented all factors except the subjective feelings of the patient. In addition to all the patients' physiological backgrounds from the well-established medical records system, we also used the subjective pain catastrophizing scale (PCS) and self-rating anxiety scale (SAS) scores recorded 1 day before surgery as baseline factors. PCS score was designed and completed by Sullivan et al. in 1995 (17, 18). The scale allowed participants to recall previous painful experiences and respond to the frequency of feelings and thoughts mentioned in the questions (19). Pain catastrophizing is a multi-dimensional structure consisting of three factors: introspection (focusing too much on pain), exaggeration (reinforcing pain to make it worse), and helplessness (thinking you can't cope with pain symptoms). The PCS assessed these three dimensions, with a total of 13 questions, each scoring from 0 (never) to 4 (always). A total score of 0 to 38 indicates that pain does not cause special pain, while a total score of 38 to 52 indicates that pain will be catastrophic. Anxiety before surgery is very common in surgical patients and the different patients always have different level of anxiety. Severe negative emotions can affect the outcome of the surgery as well as the patient's prognosis and recovery but are always easy to be ignored before surgery. The SAS scale was developed by Zung in 1971 to access the subjective feelings of patients with anxiety (20). There are 20 symptomatic factors in SAS. SAS mainly evaluates the frequency of symptoms based on the project definition. It is divided into four levels: no time or little time, little time, quite a lot of time, most of time or all of time. The score of >50, 50 to 59, 60 to 69 and >69 is considered to have symptoms of anxiety, mild anxiety, moderate anxiety, severe anxiety, respectively.

A total of 102 gynecological patients who received surgical treatment in the First Affiliated Hospital of Guangxi Medical University from January 2020 to December 2020 agreed to participate in this study. The results showed that the NRS score was correlated with EQ-5D score and changes of both EQ-5D and NRS scores meet the law of natural postoperative rehabilitation along with the time. The NRS score (Active state) was the principal influencing factor of EQ-5D score. We then divided the patients into groups according to whether they had active pain on the 30th day after surgery. As expected, patients in the pain group had a later time to get out of bed and eat, a higher incidence of postoperative complications, and a longer postoperative hospital stay. Similarly, patients in the pain group had worse EQ-5D scores. Although our data are limited, it may provide a good reference and guidance for optimizing postoperative pain management in patients with uterine malignancies.

## Materials and methods

### Patients

Patients in the Department of Gynecology who underwent surgical treatment in the First Affiliated Hospital of Guangxi Medical University from January 2020 to December 2020 were selected. Inclusion criteria: Patients age 18 years and above; American Society of Anesthesiologists (ASA) physical status 1 and 2; No communication barriers, can express their feelings in language; Informed consent can be signed. Exclusion criteria: Pre-existing chronic pain conditions; Suffering from a diagnosed mental illness; Patients with a history of use of analgesics, psychotropic drugs and antiepileptic drugs; Patients admitted to ICU after surgery. In order to ensure the authenticity of the data, the investigator did not participate in anesthesia and perioperative treatment of patients, and the investigator was blinded to anesthesia and perioperative treatment.

### Study protocol

After obtaining approval from the Ethics Committee of the first affiliated hospital of Guangxi Medical University and written consent from the selected patients, we enrolled 102 elective surgery patients. The study was registered in the Chinese Clinical Trial Registry (ChiCTR2000032759). This study was conducted in accordance with the Declaration of Helsinki. All participants were informed about the purpose of the trial.

The selected patients were visited one day before surgery (POD-1), and the patients were invited to participate in the project questionnaire, sign the informed consent, and complete the preoperative questionnaires (PCS score, SAS score and EQ-5D score). Postoperative surveys (NRS scale, EQ-5D score) were conducted on postoperative day 1 (POD1), day 3 (POD3), day 7 (POD7), day 14 (POD14), and day 30 (POD30). All patients were followed up by a face-to-face questionnaire during hospitalization and by telephone after discharge. Follow-up was terminated on postoperative day 30.

All patients were fasted for 8 h. Only clear liquids were allowed up to 2 h before the induction of anesthesia. After entering the operation room, the noninvasive blood pressure, electrocardiogram, and peripheral capillary oxygen saturation (SpO_2_) were monitored. All patients were given general anesthesia with or without nerve block. Inhalation anesthetics, intravenous anesthetics, analgesics and muscle relaxants can be selected arbitrarily according to the patient's condition and the habits of anesthesiologist to complete anesthesia induction and maintenance. During the operation, cardiovascular active drugs were used to maintain the heart rate of no less than 50 beats/min, no more than 100 beats/min, and the blood pressure was within ±20% of the preoperative blood pressure to maintain the stability of the patient's circulatory system. At the end of the anesthesia, appropriate amount of analgesics should be administered in lieu of analgesia, and a postoperative analgesia pump should be used according to the patient's will. Follow-up was performed by an investigator who was not involved in anesthesia and perioperative treatment. The investigator was blinded to anesthesia and perioperative treatment.

### Measurements

The primary data of this study was NRS and EQ-5D scores, i.e., postoperative APSP and HRQoL, at five postoperative time points. Other data were collected including Age, BMI (Body Mass Index), Education level, Charlson comorbidity index, PCS score, SAS score, Preoperative albumin, Anesthesia, Surgical methods, Operative duration, Time of first getting out of bed after surgery, Time of first eating after surgery, Postoperative analgesic pump use, Postoperative analgesic drug use, Postoperative hospital stay and Postoperative complications from the medical record system of the hospital or follow-up. The primary outcome was a comparison of postoperative rehabilitation between the pain and non-pain groups and an analysis of improvement factors.

### Sample size

According to previous studies, there are more than ten factors affecting postoperative HRQoL. In general, the sample size should not be less than 5–20 times the number of risk factors. With 16 risk factors, the sample size should be at least 80. The loss to follow-up rate is expected to be 20%, so the total sample size is estimated to be approximately 96.

### Statistical methods

All statistical analyses were performed with IBM SPSS 26.0 statistical software. All data are expressed as numbers (%) or the mean ±SD (standard deviation). Spearman's rank correlation coefficient analysis was used to analyze the correlation between NRS score or other factors and EQ-5D score after operation. T-test was used to compare the measured data between the two groups. Chi-square test was used to compare the count data of the two groups. A *p*-values of 0.05 were defined as statistically significant.

## Outcome

### Data information

**Figure 1:**
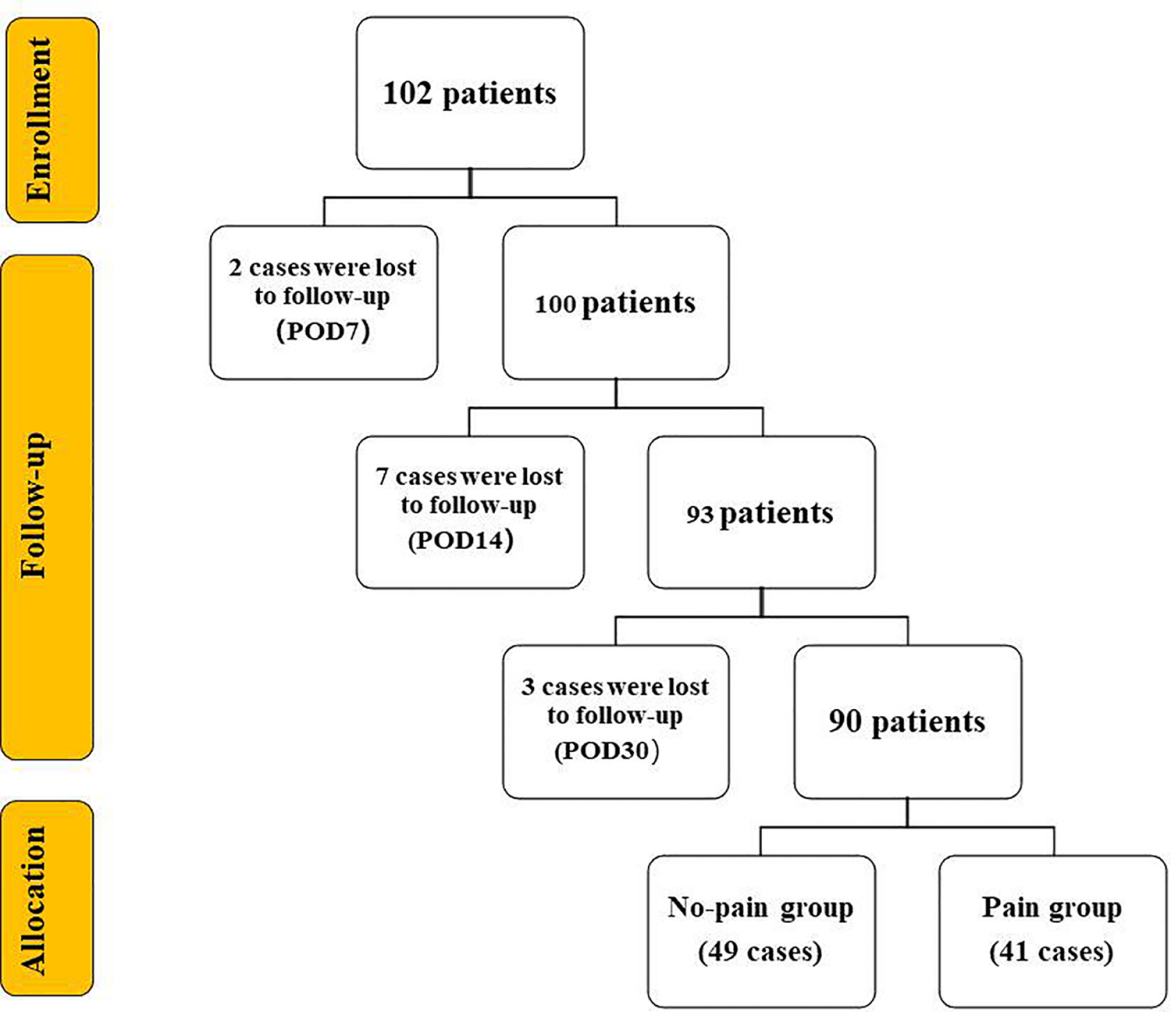
Flow chart. The number of patients decreased with the extension of follow-up. A total of 90 patients completed all follow-up on the 30th day after surgery. They were divided into two groups according to whether they had active pain or not. POD = Postoperative Day.

A total of 102 patients participated in this study according to inclusion and exclusion criteria (Figure 1). Among them, 2 patients were lost to follow-up on the 7th day after surgery, 7 patients were lost to follow-up on the 14th day after surgery, and 2 patients were lost to follow-up on the 30th day after surgery. 90 patients completed all follow-up. At postoperative day 30, 49 patients had no pain, 40 patients had mild pain at the time of activity (NRS 1–3 points), and 1 patient had moderate pain at the time of activity (NRS 4 points). Patients were divided into two groups based on pain. All patients' valid data were included in the statistical analysis.

### Changes of NRS and EQ-5D scores after surgery

We established a double-standard evaluation system by taking advantage of two well established evaluation protocols, NRS and EQ-5D, as the standard to evaluate the acute postoperative pain (APSP) and health-related quality of life (HRQoL) at day 1 before and day 1,3,7,14, and 30 after surgery (POD), respectively.

**Figure 2:**
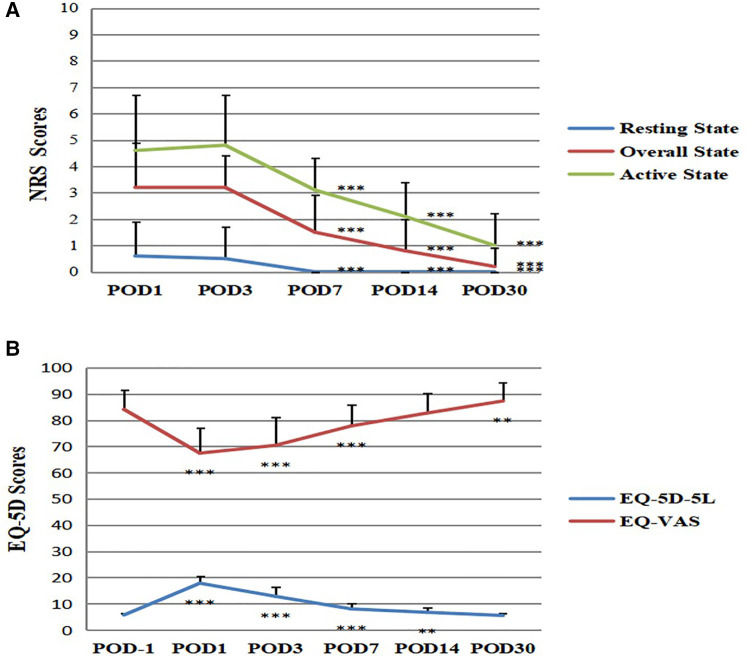
Changes of NRS and EQ-5D scores after surgery. (**A**) Changes of NRS scores after surgery. Compared with POD1, ***P < 0.001, the difference was statistically significant. (**B**) Changes of EQ-5D scores after surgery. Compared with POD-1, **P < 0.01, ***P < 0.001, the difference was statistically significant. POD = Postoperative Day.

The NRS scores include the resting state, active state, and the average of the two states, namely overall state score. The results showed that all the three NRS scores gradually decreased from POD1 to POD30, which means the APSP level decreased as time goes on after surgery (*P* < 0.001) (Figure 2A). We also found that the NRS active state scores always higher than the resting state scores at all the time point respectively (Figure 2A). The NRS scores in each group were similar on the 1st and 3rd day after surgery (*P* > 0.05), and decreased significantly on and after the 7th day (*P* < 0.001).

We first recorded the EQ-5D-5L and EQ-VAS scores of the patients one day before surgery (POD -1), as the reference of non-surgery state HRQoL level (Figure 2B). The results showed that the EQ-5D-5L scores rapidly increased from POD −1 to POD 1, and gradually decreased from POD1 to POD30, with the final score like POD −1 (*F* = 470.453, *P* < 0.001) (Figure 2B). The EQ-VAS scores rapidly decreased from POD −1 to POD 1, and gradually increased from POD1 to POD30, with the final score like POD −1 (*F* = 84.329, *P* < 0.001) (Figure 2B). This result means that the HRQoL level was rapidly decreased at POD1 and gradually increased to the non-surgery state level at POD30.

### Correlation of NRS and EQ-5D scores after surgery

To analyze the correlation between the NRS and EQ-5D scores. Spearman's rank correlation coefficient analysis was used to compare NRS scores with EQ-5D-5L and EQ-VAS scores at each time point (Tables 1, 2). The results showed that NRS scores correlated with EQ-5D-5L scores at each time point except NRS scores at 7, 14 and 30 days at rest. The correlation coefficient (r_s_) ranged from the lowest 0.465 to the highest 0.822, showing a positive correlation from moderate to strong (*P* < 0.01, Table 1). NRS scores correlated with EQ-VAS scores at each time point except NRS scores at 7, 14 and 30 days at rest. The correlation coefficient (r_s_) ranged from the lowest −0.235 to the highest −0.535, showing a negative correlation from weak to moderate (*P* < 0.01, Table 2). This means that patients with a higher APSP level always comes with a lower HRQoL level. Taken together, all these results showed that the changes of NRS and EQ-5D and the correlation of these two scores consist with the general acknowledge about APSP and HRQoL level after surgery.

**Table 1:** Correlation of NRS and EQ-5D-5L scores after surgery.

	POD1		POD3		POD7		POD14		POD30	
NRS scores	*r*_s_	*P*	*r*_s_	*P*	*r*_s_	*P*	*r*_s_	*P*	*r*_s_	*P*
Resting state	0.565	<0.01	0.465	<0.01						
Overall state	0.708	<0.01	0.822	<0.01	0.797	<0.01	0.595	<0.01	0.539	<0.01
Active state	0.712	<0.01	0.793	<0.01	0.764	<0.01	0.697	<0.01	0.756	<0.01

*P* < 0.01 represents a significant correlation. *r*_s_ is positive for positive correlation. POD = Postoperative Day.

**Table 2:** Correlation of NRS and EQ-VAS scores after surgery.

	POD1		POD3		POD7		POD14		POD30	
NRS scores	*r*_s_	*P*	*r*_s_	*P*	*r*_s_	*P*	*r*_s_	*P*	*r*_s_	*P*
Resting state	−0.400	<0.01	−0.235	<0.01						
Overall state	−0.535	<0.01	−0.474	<0.01	−0.405	<0.01	−0.433	<0.01	−0.371	<0.01
Active state	−0.529	<0.01	−0.527	<0.01	−0.421	<0.01	−0.411	<0.01	−0.473	<0.01

*P* < 0.01 represents a significant correlation. *r*_s_ is negative for negative correlation. POD = Postoperative Day.

### Analysis of related factors with EQ-5D score at 7th and 30th days after surgery

In addition to the patient's basic background, including Age, BMI (Body Mass Index) and Education level, we also included perioperative factors. We selected 13 perioperative factors including: Charlson comorbidity index, PCS score, SAS score, Preoperative albumin, Anesthesia, Surgical methods, Operative duration, Time of first getting out of bed after surgery, Time of first eating after surgery, Postoperative analgesic pump use, Postoperative analgesic drug use, Postoperative hospital stay and Postoperative complications, as the candidate factors. The EQ-5D score consists of two parts: the EQ-5D-5L and the EQ-VAS. The correlation between these 16 factors and EQ-5D-5L and EQ-VAS was analyzed at 7th and 30th days after surgery. r_s_ is positive for positive correlation and r_s_ is negative for negative correlation (Tables 3, 4). The results showed that Surgical methods, Operative duration, Time of first getting out of bed after surgery, Postoperative hospital stay and Postoperative complications were positively correlated with EQ-5D-5L score (*P* < 0.01), while PCS score, Surgical methods and Postoperative analgesic pump use were negatively correlated with EQ-VAS score on the 7th postoperative day (*P* < 0.05, Table 3). This means that the patients with open surgery, long operative time, late getting out of bed, long postoperative hospital stay and postoperative complications had higher EQ-5D-5L scores, and the patients with higher PCS scores, open surgery and postoperative analgesia pump had lower EQ-VAS scores. These patients all had poor HRQoL. The results showed that Surgical methods, Time of first getting out of bed after surgery, Postoperative hospital stay and Postoperative complications were positively correlated with EQ-5D-5L score (*P* < 0.01), Education level and Preoperative albumin were positively correlated with EQ-VAS score (*P* < 0.05), while Surgical methods, Time of first getting out of bed after surgery, Postoperative analgesic pump use and Postoperative complications were negatively correlated with EQ-VAS score on the 30th postoperative day (*P* < 0.05, Table 4). This means that the patients with open surgery, late getting out of bed, long postoperative hospital stay and postoperative complications had higher EQ-5D-5L scores, the patients with lower education level and preoperative albumin had lower EQ-VAS scores, and the patients with open surgery, late getting out of bed, postoperative analgesia pump and Postoperative complications had lower EQ-VAS scores. These patients all had poor HRQoL.

**Table 3:** Analysis of related factors with EQ-5D score at 7th day after surgery.

	Relevant Factors	*r*_s_	*P*
EQ-5D-5L	Surgical methods	0.605	<0.001***
(POD7)	Operative duration	0.278	0.005**
	Time of first getting out of bed after surgery	0.324	0.001***
	Postoperative hospital stay	0.551	<0.001***
	Postoperative complications	0.356	<0.001***
EQ-VAS	PCS score	−0.220	0.028*
(POD7)	Surgical methods	−0.268	0.007**
	Postoperative analgesic pump use	−0.218	0.030*

* *P* < 0.05.

** *P* < 0.01.

*** *P* < 0.001 has correlation. *r*_s_ is positive for positive correlation and *r*_s_ is negative for negative correlation. POD = Postoperative Day.

**Table 4:** Analysis of related factors with EQ-5D score at 30th day after surgery.

	Relevant Factors	*r*_s_	*P*
EQ-5D-5L	Surgical methods	0.593	<0.001***
(POD30)	Time of first getting out of bed after surgery	0.314	0.003**
	Postoperative hospital stay	0.498	<0.001***
	Postoperative complications	0.400	<0.001***
EQ-VAS	Education level	0.211	0.046*
(POD30)	Preoperative albumin	0.227	0.032*
	Surgical methods	−0.346	0.001**
	Time of first getting out of bed after surgery	−0.215	0.042*
	Postoperative analgesic pump use	−0.236	0.025*
	Postoperative complications	−0.217	0.040*

* *P* < 0.05.

** *P* < 0.01.

*** *P* < 0.001 has correlation. *r*_s_ is positive for positive correlation and *r*_s_ is negative for negative correlation. POD = Postoperative Day.

### Analysis of principal factors affecting EQ-5D score at 7th and 30th days after surgery

Multivariate linear regression analysis was conducted on the factors related to EQ-5D-5L and EQ-VAS scores at 7th and 30th days after surgery (Tables 5, 6). On the 7th postoperative day, the dependent variable was EQ-5D-5L score, the predictive variable was constant, Surgical methods, Operative duration, Time of first getting out of bed after surgery, Postoperative hospital stay, Postoperative complications, NRS score (Overall state) and NRS score (Active state) (Table 5). The results show that the regression equation is significant, *F* = 35.734, *P* < 0.001. Postoperative hospital stay (*β *= 0.186, *P* = 0.009), NRS score (Overall state) (*β *= 0.412, *P* < 0.001) and NRS score (Active state) (*β *= 0.298, *P* < 0.001) significantly positively predicted EQ-5D-5L score. These variables accounted for 71.1% of the variation in EQ-5D-5L score (*R*^2 ^= 0.711). On the 30th postoperative day, the dependent variable was EQ-5D-5L score, the predictive variable was constant, Surgical methods, Time of first getting out of bed after surgery, Postoperative hospital stay, Postoperative complications, NRS score (Overall state) and NRS score (Active state) (Table 5). The results show that the regression equation is significant, *F* = 34.028, *P* < 0.001. Postoperative hospital stay (*β *= 0.237, *P* = 0.004), NRS score (Overall state) (*β *= 0.406, *P* < 0.001) and NRS score (Active state) (*β *= 0.388, *P* < 0.001) significantly positively predicted EQ-5D-5L score. These variables accounted for 69.0% of the variation in EQ-5D-5L score (*R*^2 ^= 0.690). On the 7th postoperative day, the dependent variable was EQ-VAS score, the predictive variable was constant, PCS score, Surgical methods, Postoperative analgesic pump use, NRS score (Overall state) and NRS score (Active state) (Table 6). The results show that the regression equation is significant, *F* = 8.386, *P* < 0.001. PCS score (*β *= −0.259, *P* = 0.005) and NRS score (Active state) (*β *= −0.380, *P* = 0.003) significantly negatively predicted EQ-VAS score. These variables accounted for 27.2% of the variation in EQ-VAS score (*R*^2 ^= 0.272). On the 30th postoperative day, the dependent variable was EQ-VAS score, the predictive variable was constant, Education level, Preoperative albumin, Surgical methods, Time of first getting out of bed after surgery, Postoperative analgesic pump use, Postoperative complications, NRS score (Overall state) and NRS score (Active state) (Table 6). The results show that the regression equation is significant, *F* = 7.191, *P* < 0.001. Education level (*β *= 0.230, *P* = 0.011) significantly positively predicted EQ-VAS score, and NRS score (Overall state) (*β *= −0.323, *P* = 0.003) and NRS score (Active state) (*β *= −0.375, *P* = 0.005) significantly negatively predicted EQ-VAS score. These variables accounted for 35.8% of the variation in EQ-VAS score (*R*^2 ^= 0.358). This means that the patients with long postoperative hospital stay and higher NRS scores (Overall state and Active state) had higher EQ-5D-5L scores, and the patients with lower education level and higher PCS and NRS scores (Overall state and Active state) had lower EQ-VAS scores. These patients all had poor HRQoL.

**Table 5:** Analysis of principal factors affecting EQ-5D-5L score at 7th and 30th days after surgery.

	Principal Factors	*β*	*P*
EQ-5D-5L(POD7)	Postoperative hospital stay	0.186	0.009*
(R^2 ^= 0.711)	NRS score (Overall state)	0.412	<0.001**
	NRS score (Active state)	0.298	<0.001**
EQ-5D-5L(POD30)	Postoperative hospital stay	0.237	0.004*
(*R*^2 ^= 0.690)	NRS score (Overall state)	0.406	<0.001**
	NRS score (Active state)	0.388	<0.001**

* *P* < 0.01.

** *P* < 0.001 has correlation. *β* is positive for positive correlation and *β* is negative for negative correlation. POD = Postoperative Day.

**Table 6:** Analysis of principal factors affecting EQ-VAS score at 7th and 30th days after surgery.

	Principal Factors	*β*	*P*
EQ-VAS (POD7)	PCS score	−0.259	0.005**
(*R*^2 ^= 0.272)	NRS score (Active state)	−0.380	0.003**
EQ-VAS (POD30)	Education level	0.230	0.011*
(*R*^2 ^= 0.358)	NRS score (Overall state)	−0.323	0.003**
	NRS score (Active state)	−0.375	0.005**

* *P* < 0.05.

** *P* < 0.01 has correlation. *β* is positive for positive correlation and *β* is negative for negative correlation. POD = Postoperative Day.

### Comparison of postoperative rehabilitation between pain and non-pain patients

On the 30th postoperative day, 90 patients completed all follow-up. According to the above, NRS score (Active state) is the principal factor affecting EQ-5D score. Therefore, patients were grouped according to whether they had active pain or not on the 30th postoperative day. There were 49 patients in the non-pain group and 41 patients in the pain group (Tables 7, 8). There was no significant difference in baseline data, including Age, BMI, Education level, Charlson comorbidity index, PCS score, SAS score and Preoperative albumin, between the two groups (*P* > 0.05, Table 7). The perioperative conditions of the two groups were compared (Table 8). There was no significant difference between the two groups in terms of Anesthesia, Operative duration, Postoperative analgesic pump use and Postoperative analgesic drug use (*P* > 0.05). There were significant differences between the two groups in terms of Surgical methods, Time of first getting out of bed after surgery, Time of first eating after surgery, Postoperative hospital stay and Postoperative complications (*P* < 0.05). The patients in the pain group had a higher rate of open surgery and postoperative complications, and they got out of bed late, ate late and stayed in hospital longer.

**Table 7:** Baseline levels were compared between the two groups.

	Non-pain group	Pain group		* *
	(*N* = 49)	(*N* = 41)	*t*/*χ*^2^	*P*
Age (years)	52 ± 9	51 ± 8	0.718	0.475
BMI	24.2 ± 4.4	23.4 ± 4.0	0.922	0.359
Education level			0.196	0.906
Elementary	17	16		
Medium	21	16		
Advanced	11	9		
Charlson comorbidity index	1.0 ± 0.9	0.8 ± 0.8	1.157	0.251
PCS score	16.1 ± 4.7	15.8 ± 5.0	0.287	0.775
SAS score	30.5 ± 9.8	31.7 ± 10.8	0.571	0.570
Preoperative albumin	38.7 ± 3.8	38.6 ± 3.8	0.157	0.876

All data are expressed as numbers (%) or the mean ± SD. *P* > 0.05, there was no significant difference.

**Table 8:** Perioperative conditions of the two groups were compared.

	Non-pain group	Pain group		
	(*N* = 49)	(*N* = 41)	*t*/χ^2^	*P*
Anesthesia			0.647	0.421
General anesthesia	40	36		
Combined anesthesia	9	5		
Surgical methods			37.631	<0.001***
Open surgery	4	29		
Laparoscopic surgery	45	12		
Operative duration (min)	222 ± 88	243 ± 65	1.286	0.202
Time of first getting out of bed after surgery (h)	28 ± 10	38 ± 16	3.654	<0.001***
Time of first eating after surgery (h)	22 ± 10	29 ± 16	2.347	0.021*
Postoperative analgesic pump use			3.096	0.079
Yes	39	38		
No	10	3		
Postoperative analgesic drug use			1.606	0.205
Yes	38	36		
No	11	5		
Postoperative hospital stay (days)	7 ± 3	11 ± 6	3.635	<0.001***
Postoperative complications			11.069	0.004**
None	37	17		
Grade I	10	18		
Grade III-b	2	6		

All data are expressed as numbers (%) or the mean ± SD.

* *P* < 0.05.

** *P* < 0.01.

*** *P* < 0.001, there was significant difference.

### Comparison of EQ-5D scores between pain and non-pain patients

**Figure 3:**
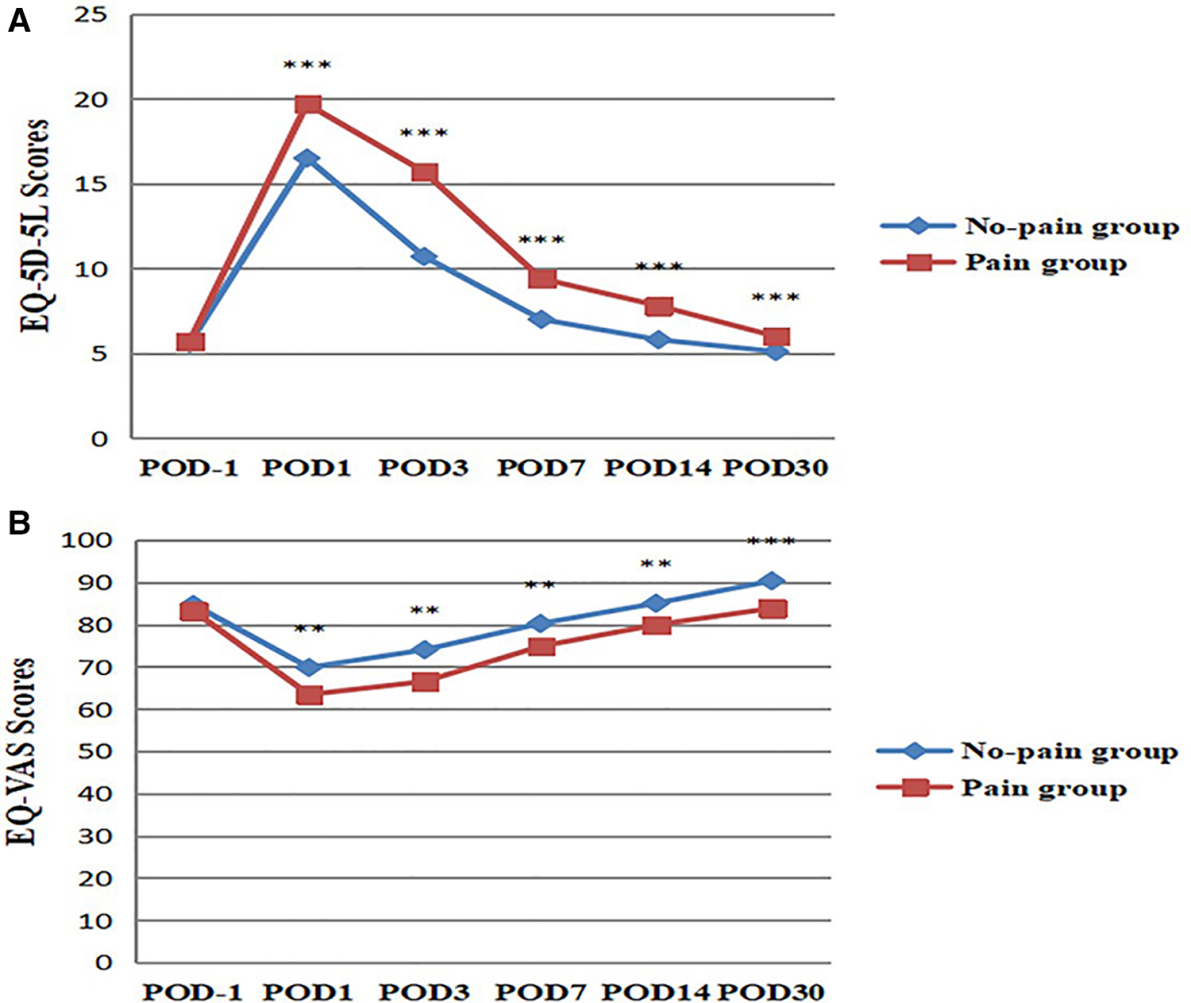
Comparison of EQ-5D scores between pain and non-pain patients. (**A**) EQ-5D-5L scores were compared between the two groups. ***P < 0.001, the difference was statistically significant. (**B**) EQ-VAS scores were compared between the two groups. **P < 0.01, ***P < 0.001, the difference was statistically significant. POD = Postoperative Day.

**Figure 4:**
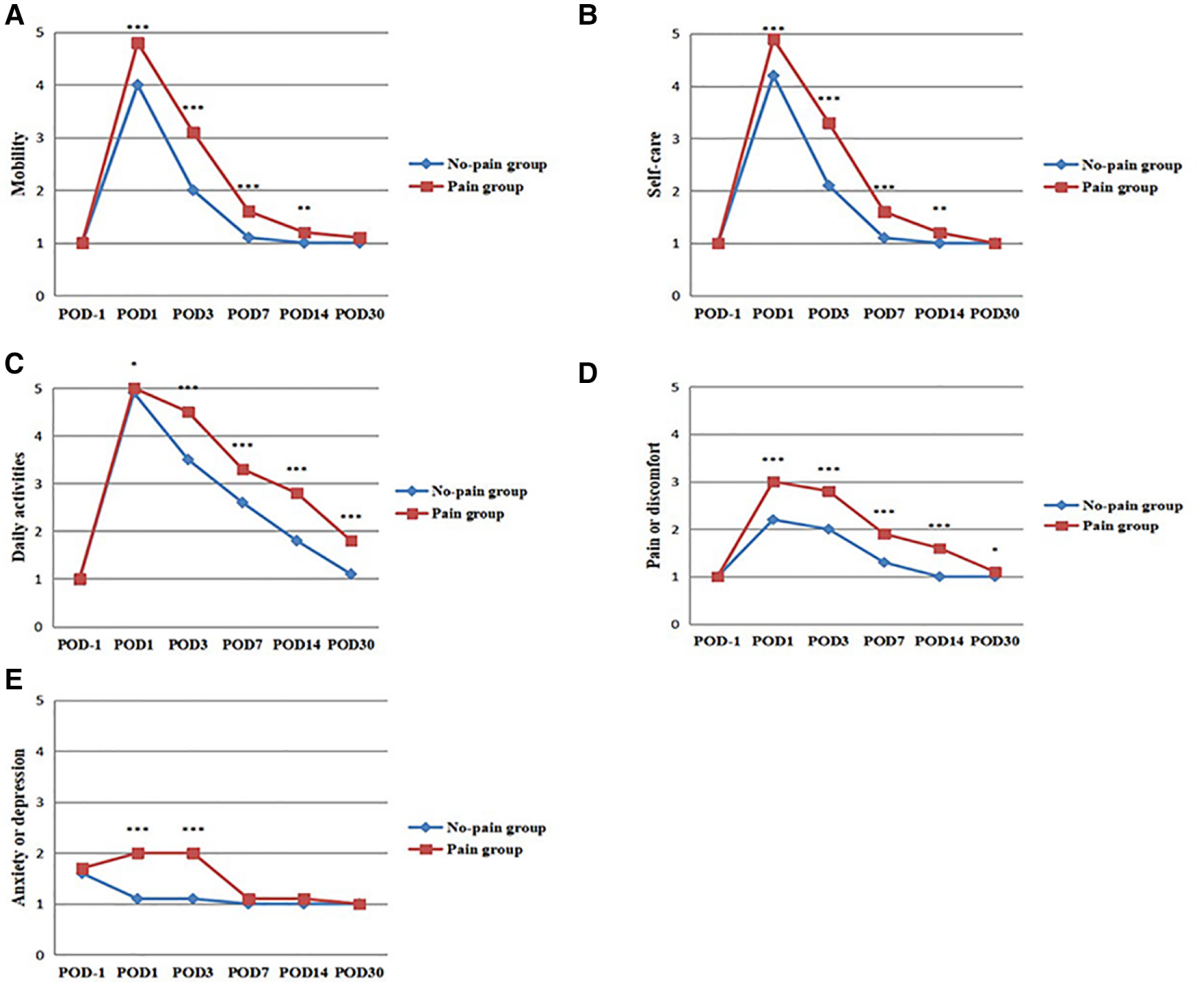
Comparison of EQ-5D-5L scores between pain and non-pain patients. (**A**) Mobility scores were compared between the two groups. (**B**) Self-care scores were compared between the two groups. (**C**) Daily activities scores were compared between the two groups. (**D**) Pain or discomfort scores were compared between the two groups. (**E**) Anxiety or depression scores were compared between the two groups. *P < 0.05, **P < 0.01, ***P < 0.001, the difference was statistically significant. POD = Postoperative Day.

EQ-5D-5L and EQ-VAS scores were compared between patients in pain and non-pain groups at day 1 before and day 1,3,7,14, and 30 after surgery (POD), respectively (Figures 3A,B). There was no significant difference in EQ-5D-5L and EQ-VAS scores between the two groups at day 1 before surgery (*P* > 0.05). This indicates that preoperative HRQoL was comparable between the two groups of patients. There were significant differences in EQ-5D-5L and EQ-VAS scores between the two groups at day 1, 3, 7, 14, and 30 after surgery (*P* < 0.01). Patients in the pain group had worse postoperative HRQoL. EQ-5D-5L has a total of five dimensions, namely mobility, self-care, daily activities, pain or discomfort, and anxiety or depression (Figures 4A–E). There was no significant difference between the two groups in five dimensions at day 1 before surgery (*P* > 0.05). In terms of mobility and self-care, the difference between the two groups was statistically significant at day 1, 3, 7, and 14 after surgery (*P* < 0.01, Figures 4A,B). In terms of daily activities and pain or discomfort, the difference between the two groups was statistically significant at day 1, 3, 7, 14, and 30 after surgery (*P* < 0.05, Figures 4C,D). In terms of anxiety or depression, the difference between the two groups was statistically significant at day 1 and 3 after surgery (*P* < 0.001, Figure 4E). The pain group had higher scores in these five dimensions and worse postoperative HRQoL.

## Discussion

With the development of medical technology, many diseases can be treated by surgical resection. Because of the large trauma and severe and persistent wound pain, it often affects the postoperative rehabilitation of patients, prolongs the length of hospitalization, increases the cost of hospitalization, and even affects the quality of life and survival of patients after discharge from hospital, increase the social and family burden. Postoperative pain and rehabilitation have also become the most concerned issues for patients undergoing surgery. The most common complaint among postoperative patients is postoperative pain, which is acute pain that usually lasts no more than 7 days due to surgery-related tissue damage (2) and can develop into chronic pain if poorly controlled (8). Severe postoperative pain leads to psychological and behavioral adverse reactions, which affects patients' daily life and is not conducive to postoperative rehabilitation, thus reducing patients' postoperative HRQoL. Severe pain may even affect the patient's daily life, such as poor quality of sleep at night, limited activities, resulting in prolonged hospital stay, increased medical costs (21). At present, there are many clinical studies on the impact of postoperative pain on rehabilitation, which mainly take postoperative hospital stay (6, 9) and postoperative complications (22, 23) as indicators, but neglect the subjective evaluation of patients. For patients, postoperative recovery should be a return to a normal level of quality of life, that is, to the patient's baseline level before surgery or to a level consistent with social norms, a process that may take weeks or months (24). HRQoL is one of the self-evaluation indexes of patients, which can directly reflect the postoperative rehabilitation quality of patients (25, 26). This paper uses EQ-5D scale as the evaluation index of HRQoL. Therefore, from the point of view of comfortable medical treatment, the combination of objective and subjective indicators can be more comprehensive evaluation of postoperative rehabilitation of patients.

At present, most of the studies focus on the hospitalization period of patients, while there are few studies on the correlation between APSP and HRQoL after discharge. Although the period from discharge to return to daily life is an important period of rehabilitation, it is also a neglected period in current clinical research (10). The development of APSP and HRQoL is very important, but most studies only focus on a single time point after surgery or only on hospitalization, rather than the entire recovery process. According to the past clinical experience, most of the patients can be discharged from the hospital at 1 week after operation and can resume normal life at 1 month after operation, therefore, these two time points were defined as two time points of postoperative recovery.

Uterine malignant tumor is a common gynecological disease in women, among which endometrial cancer and cervical cancer are more common, mostly seen in women over 45 years old. Because of the improvement of medical technology and People's awareness, the early detection rate of uterine malignant tumor has also increased. Patients can get early diagnosis and treatment, and the case fatality rate is reduced. Therefore, more patients with uterine malignant tumors need surgical treatment. The study on the patients with uterine malignant tumor can improve the perioperative scheme of these patients in a short time and enlarge the range of patients who can benefit from it. In this study, the peak period of postoperative pain was 1–3 days after surgery. After 3 days, the pain score continued to decline, consistent with the conclusion that the peak of acute pain occurred between 24 and 48 h postoperatively. With the extension of postoperative time, the EQ-5D-5L score of patients decreased gradually, and the EQ-VAS score increased gradually. The lower the EQ-5D-5L score and the higher the EQ-VAS score, the better the health status of the patients. It indicates that the pain of patients is gradually relieved and HRQOL is getting better and better. HRQoL can reach or even exceed the preoperative level on the 30th day after surgery. These conditions were in accordance with the clinical rules. Patients' NRS scores were significantly correlated with EQ-5D-5L and EQ-VAS scores in HRQoL evaluation. Both fatigue and pain symptoms had a negative effect on HRQoL, which was consistent with postoperative patients (27). Previous studies have shown that increased postoperative pain reduces postoperative HRQoL (2). This is consistent with this study. Another study showed that good postoperative analgesia reduced procedure-related pain 1 month after surgery and improved HRQoL 3 months after surgery (28).

On the 7th and 30th days after surgery, there are also some perioperative factors related to EQ-5D score. Therefore, the principal factors affecting EQ-5D-5L score were postoperative hospital stay, NRS score (Overall state) and NRS score (Active state), which were positively correlated with EQ-5D-5L score. Education level, PCS score, NRS score (Overall state) and NRS score (Active state) were the principal factors affecting EQ-VAS score, among which Education level was positively correlated with EQ-VAS score and others were negatively correlated with EQ-VAS score. The lower the education level, the longer the hospital stay, the higher the PCS and NRS scores, the worse the HRQoL was. Therefore, EQ-5D score, especially EQ-5D-5L score, can be used for postoperative rehabilitation evaluation of patients as a subjective indicator.

On the 30th day after surgery, 41 of 90 patients had active pain, the incidence of pain was 46%. A survey in the United States shows that the incidence of moderate or severe pain after discharge is as high as 74% (3). Therefore, postoperative pain, especially post-discharge pain, should be brought to the attention of clinicians. Patients were divided into two groups according to whether they had pain on the 30th day after surgery. Compared with the two groups of patients, patients in the pain group had a later time to get out of bed and eat, a higher incidence of postoperative complications, and a longer postoperative hospital stay. The patients in the pain group tend to have worse EQ-5D-5L and EQ-VAS scores, in which EQ-5D-5L scores include mobility, self-care, daily activities, pain or discomfort and anxiety or depression. Therefore, from the subjective and objective point of view, postoperative pain can lead to delayed rehabilitation of patients after surgery. EQ-5D score can be used to evaluate the rehabilitation of patients after operation. Endoscopic surgery can reduce the postoperative pain of patients and improve the rehabilitation quality of patients.

The concept of Enhanced Recovery after Surgery (ERAS) has been widely used in clinical practice. ERAS refers to the application of a series of treatment strategies based on evidence-based medicine through multidisciplinary collaboration to reduce surgical stress, postoperative pain, and postoperative complications, to promote postoperative recovery of patients (29). Analyzing the influence of postoperative pain on postoperative rehabilitation can provide data support for improving the postoperative analgesia management of gynecological patients. This also provides a basis for taking relevant measures to promote the rehabilitation of patients. In this study, minimally invasive surgery can be used as a perioperative program to improve the pain of patients after discharge and promote postoperative rehabilitation. Studies have shown that patients undergoing orthopaedic, thoracic and open surgery have higher postoperative pain scores than patients undergoing other types of surgery (30). Minimally invasive surgery, such as endoscopic surgery, is very mature (31). Patients can choose minimally invasive surgery whenever possible. Pain treatment not only requires accurate results and fewer side effects but also emphasizes personalized analgesia based on the disease, to reduce the amount of each drug and the corresponding side effects, thus accelerating the rapid recovery of patients and reducing the length of hospital stay and medical costs (32). We should pay more attention to the patients’ self-feelings, combined with clinical indicators, to promote postoperative recovery of patients. In future studies, the protocol may serve as a basis for prospective evaluation of patients in other departments and facilitate postoperative rehabilitation of other types of patients.

## Conclusions

The postoperative rehabilitation of patients in the pain group was poor. Minimally invasive surgery can reduce postoperative pain and promote postoperative rehabilitation. EQ-5D score can be used as a subjective index to evaluate postoperative rehabilitation.

## Data Availability

Publicly available datasets were analyzed in this study. This data can be found here: The data supporting the findings of this study are available from the corresponding author on reasonable request. xybdoctor@163.com.
